# Validation of nursing documentation regarding in-hospital falls: a cohort study

**DOI:** 10.1186/s12912-021-00577-4

**Published:** 2021-04-09

**Authors:** Karolina Krakau, Helene Andersson, Åsa Franzén Dahlin, Louise Egberg, Eila Sterner, Maria Unbeck

**Affiliations:** 1 Department of Rehabilitation Medicine, Danderyd Hospital, Danderyd, Sweden; 2 Department of Clinical Sciences, Danderyd Hospital, Karolinska Institutet, Solna, Sweden; 3 Department of Infection, Danderyd Hospital, Danderyd, Sweden; 4 Department of Neurology, Danderyd Hospital, Danderyd, Sweden; 5 Department of Surgery and Urology, Danderyd Hospital, Danderyd, Sweden; 6 Department of Neurobiology, Care Sciences and Society, Karolinska Institutet, Solna, Sweden; 7 Department of Trauma, Acute Care Surgery and Orthopedics, Karolinska University Hospital, Solna, Sweden; 8 School of Education, Health and Social Studies, Dalarna University, Falun, Sweden

**Keywords:** Documentation, Fall, Nursing, Patient safety, Quality indicator

## Abstract

**Background:**

In-hospital fall incidents are common and sensitive to nursing care. It is therefore important to have easy access to valid patient data to evaluate and follow-up nursing care. The aim of the study was to validate the nursing documentation, using a specific term in the registered nurses´ (RNs´) discharge note, regarding inpatient falls according to the outcome of a digitalized data extraction tool and the discharge note itself.

**Methods:**

At a teaching hospital, 31,571 episodes of care were eligible for inclusion in this retrospective cohort study. A stratified sampling including five groups was used, two with random sampling and three with total sampling. In total, 1232 episodes of care were reviewed in the electronic patient record using a study-specific protocol. Descriptive statistics were used.

**Results:**

In total, 590 episodes of care in the study cohort included 714 falls. When adjusted for the stratified sampling the cumulative incidence for the study population was 1.9%.

The positive predictive value in total for the data extraction tool regarding the presence of any fall, in comparison with the record review, was 87.4%. Discrepancies found were, for example, that the RNs, at discharge, stated that the patient had fallen but no documented evidence of that could be detected during admission. It could also be the opposite, that the RNs correctly had documented that no fall had occurred, but the data extraction tool made an incorrect selection. When the latter had been withdrawn, the positive predictive value was 91.5%.

Information about minor injuries due to the fall was less accurate. In the group where RNs had stated that the patient had fallen without injury, minor injuries had actually occurred in 28.3% of the episodes of care.

**Conclusions:**

The use of a specific term regarding fall in the RNs´ discharge note seems to be a valid and reliable data measurement and can be used continuously to evaluate and follow-up nursing care.

## Background

Falls and their consequences pose a large health problem in society and its frequency increases with age [1]. In 2017 in Sweden, almost 67,000 people (around 662 individuals per 100,000 inhabitants) were hospitalized due to falls and seven out of ten were older than 65 years [2]. In acute hospital care, the numbers of potential fallers are consequently high, as almost half of the patients are over 65 years old. However, age is hardly the only contributor to falls. The consequences of the injury/illness of the person – its impact on motor, sensory and cognitive function, the side effects of treatments, and the unfamiliar environment, add up to a much more complex situation. Depending on the setting, whether it is short- or long-term care, which diagnoses, and other risk factors dominate; in-hospital fall rates vary from approximately 1.7 to 16.9 falls per 1000 patient days [3–9]. However, the information on the number of in-hospital falls, essential information to enable evaluation of different patient safety measures, have not always been accessible in the clinical work.

In 2012, the Nursing Board in Stockholm County Council was assigned to develop relevant quality indicators sensitive to nursing care that could be used in acute hospitals´ follow-up of falls. Process indicators were already in place or progress at most of the acute hospitals and focused on whether the risk assessments were made in accordance with directives and if preventive actions were taken for individuals at risk of falling. An outcome indicator measuring the proportion of patients falling during a current care episode, in total and those with injury, was considered adequate to reflect the fall frequency and quality of nursing inpatient care.

For measurements of this new indicator, a continuous data collection method was recommended by the Nursing Board. The electronic patient record (EPR) was concluded to be the safest data collection source, as incident reporting systems often lack consistency [10–12] and about 25% of falls are left unreported [13]. However, a structured way to document falls in the patient’s record was not in place at that time and needed to be created. A new term,” falls during current episode of care” with three predefined answers, was therefore added to the inpatient registered nurses (RNs) discharge note. An episode of care is the time frame of which a specific provider of care is responsible for the care of the patient. The predefined answers in the term were no falls during current episode of care, has fallen without injury or has fallen with injury. The term was tested and gradually implemented in the four major acute hospitals of Stockholm County Council from January 2013 and forward.

It is essential to have easy access to valid patient data and therefore a well-kept patient record is the basis to timely evaluate and follow-up nursing care. Thus, when a quality indicator or improvement strategies rely on the patient record to guide and improve patient safety, the accuracy of nursing documentation as well as how the data is extracted from the record becomes extra crucial. The implementation of a fall term in an EPR is a standardized method to identify continuous outcome data regarding inpatient falls, but its validity, from both nursing documentation and data extraction perspective, needs to be evaluated and we found a knowledge gap in the literature regarding this.

The aim of the study was to validate the nursing documentation, using a specific term in the RNs´ discharge note, regarding inpatient falls according to the outcome of a digitalized data extraction tool and the discharge note itself.

## Methods

### Study design

This was a retrospective cohort study using a structured record review, patient data via a digitalized data extraction tool, and clinical incident reports.

### Setting

The study was carried out at a teaching hospital in a metropolitan area in Sweden. In 2016, where this study’s sample groups origin from, the hospital had around 420 beds and a catchment area of approximately 500,000 inhabitants. Around 48,000 inpatient episodes of care were executed, divided into different kinds of medical and surgical wards, as well as one intensive care unit and one delivery ward. A total of 410,000 out-patient visits were carried out, and the hospital had around 3800 employees.

### Definitions

#### Fall

The national definition [14], based on the World Health Organization, was used: “a fall is an event when a patient unintentionally ends up on the floor or ground, irrespectively if an injury occurs or not”. This means that it is not only when someone stumbles or slips that is considered a fall, but also when someone rolls out of bed or slides down the floor from a chair.

#### Episode of inpatient care

The episode of inpatient care, herby named episode of care in this study, reflects the organization of acute health care at Stockholm County Council in 2016, and is described as follows:

An *episode of care* is the time frame of which a specific provider of care is responsible for the care of the patient. It is initiated by a referral and holds the time from admission to a ward to discharge. The episode of care is either acute or planned but does not include the time at the Emergency Department. Transferals between wards at the hospital may occur within this time frame, for example, due to the need for advanced intensive care or, more often, due to bed occupancy. As the responsibility of care still lies on the original provider of care, this “at a distance care” is included in the ongoing episode of care. However, when the medical problems of the patient shift focus (i.e. the acute heart condition is under control while the moderate pancreatitis remains untreated) or if the patient needs to change hospital, this transferal is considered a discharge – the episode of care ends and a new one begins.

### Nursing documentation routines and implementation process

At each discharge, a discharge note is used to recapitulate the episode of care. The template for this in the EPR contains predefined terms and sometimes even predefined answers to select from to help the RN to summarize the information on the patient’s care, the current risks, and the care plan needed ahead in a holistic and structured way. Some terms are mandatory, while some are not.

In January 2013, the new term “falls during current episode of care” was added to this discharge note, first at one inpatient unit where the Fall Prevention Board of the present hospital tested its implementation. The Quality Board of the hospital, where representatives from all inpatient units were included, decided that all inpatient units except the Gynecology and Obstetrics Department should use this term. The Nursing Informatic Board was responsible for gradually adding the term to all discharge note templates (completed in May 2013), that it was made mandatory (completed in September 2013) and that instructions on how to document on this term were delivered to the units (most of the units were represented in the Nursing Informatic Board).

By making it mandatory, a reminder emerged automatically on the screen if documentation had been left out in the discharge note and to exit the discharge note without any documentation on this term, this standpoint needed to be signed by the RN.

The instructions on how to document on the term “falls during current episode of care” was to always use one of the predefined answers;

1. No falls during current episode of care
2. Has fallen without injury
3. Has fallen with injury

If the patient had fallen more than once during the episode of care, it was the worst outcome of the falls that should be noted. Text answers could always be added to the predefined answer but should not be used solely as only the predefined answers could be traced digitally.

The Fall Prevention Board checked and reported on the coverage ratio of using the term and its predefined answers. It increased with a satisfying rate, from 8.3 to 96.3% within the first year and the completeness of documentation of the term has remained stable since then. Also, when the outcome of falls was reported to the units, a reminder of the definition of falls and the definition of injury due to falls were repeated. There were no instructions given on how to define an episode of care, implying it was common knowledge.

### Inclusion and exclusion criteria of episodes of care, sampling strategy, data extraction and power calculation

All episodes of care, both acute and elective, with a discharge date during 2016 (*n* = 31,571) were eligible for inclusion. Episodes of care from the following departments; surgical, urological, orthopedic, medical, renal medicine, rehabilitation medicine, infection and cardiology were included in the study. The Gynecology and Obstetrics Department were not included since they did not use the present term.

The Business Intelligence tool, QlikView®, was used by the hospital to extract and visualize administrative, episode of care and clinical patient data to underly for analyses and decisions. The new fall term with structured data enabled the hospital to have fall outcome data on all episodes of care, instead of manually collected cross-sectional data. However, when using a digitalized extraction data tool, the extracted data must be validated. To do this we used a stratified sampling including five sample groups (Table 1).

**Table 1:** Sample groups, selection criteria and numbers of episodes of care in total and reviewed

Group	Selection criteria for each episode of care during 2016 when using a data extraction tool	Total episodes of care, study populations N	Reviewed episodes of care, study sample groups n (%)
A	• No nursing discharge note is used	98	98 (100)
B	• Nursing discharge note is used • No documentation on the term “falls during current episode of care”	310	233 (75.2)
C	• Nursing discharge note is used • The term “falls during current episode of care” has been used • The predefined answer *“No falls”* has been used	30,495	233 (0.8)
D	• Nursing discharge note is used • The term “falls during current episode of care” has been used • The predefined answer *“Has fallen without injury”* has been used	499	499 (100)
E	• Nursing discharge note is used • The term “falls during current episode of care” has been used • The predefined answer *“Has fallen with injury”* has been used	169	169 (100)
Total		31,571	1232 (3.9)

In earlier in-hospital quality measurements a three-percentage points difference was found between the RNs´ documentation using the term “falls during current episode of care” in the discharge note (2%) compared to the documentation made throughout the whole episode of care and by all professionals (5%). The sample groups B and C consisted of 233 randomly selected episodes of care, respectively, according to a power calculation, 80% power and a 95% confidence level with a 2.19% margin of error, to detect the three-percentage points difference. All episodes of care from the sample groups A, D and E were included.

### Data sources and collection methods

#### Review protocol, education, and calibration among the reviewers

All authors were included in the study design process and the development of the study-specific review protocol with its instructions - the latter made in an iterative process. Five test records were reviewed independently, and a separate meeting was held where the reviewers reported all their findings and classifications according to the review protocol and discussions were held until consensus was reached. The six authors, all RNs with a PhD and experience of structured record review, served as reviewers.

#### Review process

The reviewers were assigned to different sample groups and read through meticulously all information related to falls from admission to discharge using all professionals´ notes in the EPR (TakeCare©). Examples of variables collected, using the review protocol, were presence of a fall, presence of an injury in relation to the fall, if the episode of care belonged to the right sample group in relation to the data extraction tool or the RNs´ actual documentation in the fall term in the discharge note, and if not, the potential cause why, and the severity of the fall [15]. Demographic data such as age, gender, type of episode of care, and length of hospital stay were collected via the patient administrative system.

Meetings were held regularly during the review process where difficulties and ambiguities were discussed. To check for reliability, two of the reviewers were assigned to carry out double reviews on every tenth episode of care per sample group in a total of 133 episodes of care.

#### Clinical incident reports

To get a hold of the number of incident reports regarding inpatient falls of the matching year and departments, the incident report system at the hospital was reviewed.

### Statistical analysis

Descriptive statistics, such as frequencies, percentages, and means were used. Cohen’s kappa was calculated for inter-rater reliability between the reviewers. Positive predictive values (A/(A + B) × 100) were used to evaluate the extraction from the data extraction tool. The RNs´ actual documentation in the fall term in the discharge note (in relation to what was found in the record review process where the documentation of the whole episode of care) was scrutinized. An analysis of the potential errors regarding fall outcome was also carried out. IBM SPSS Statistics version 25 (IBM Inc., USA) was used to calculate the results.

## Results

### Fall incidence

In total, 1232 (3.9%) episodes of care of the 31,571 eligible were reviewed. Almost half of these contained at least one in-hospital fall (*n =* 590, 47.9%). When adjusted for the random sampling process for groups B and C, the cumulative incidence was 1.9% regarding episodes of care with at least one fall for the study population, i.e. the included departments during 2016. The total number of falls was 714, as some patients fell more than one time.

### Demographics

Elderly and acute admissions were common in the study cohort. Men were also more represented than women and the majority stayed less than 1 week at the wards (Table 2).

**Table 2:** Demographics for 1232 patients and episodes of care

Sex, n (%)	
Men	677 (55.0)
Women	555 (45.0)
Age, median age in years (range)	74 (15–102)
Type of admission, n (%)	
Acute admission	1046 (84.9%)
Elective admission	179 (14.5%)
Unknown	7 (0.6%)
Length of stay, median days (range)	5 (1–117)

### Specific findings in relation to the sample groups

According to the data extraction tool, the term “falls during current episode of care” had been used in the RNs´ discharge note in 98.7% of the 31,571 episodes of care.

In the EPRs where the fall term had not been used (groups A and B), falls were detected during review process in 3.1% (3/98) of the episodes of care in group A and in 1.3% (3/233) in group B. Noteworthy, 15 patients in group A and 24 patients in group B had deceased during the episode of care, none caused by falls, but a factor that limits the RNs´ documentation at discharge.

In the EPRs where the predefined answer “No falls” had been used (group C), no falls were identified in the randomized sample. The positive predictive value regarding presence of a fall for this group was 0%, and this was accurate since there was not supposed to be any falls in this group. On the other hand, the negative predictive value was 100% for the record review, and the data extraction tool, as well as the RNs´ actual documentation in the fall term.

In the EPRs where the predefined answer “Has fallen without injury” was used (group D), according to the data extraction tool, 11.6% episodes of care (58/499), the reviewers found no trace of any fall, and in 18 (31.0%) of these, the RNs also had correctly used the predefined answer, “no falls during current episode of care.” The latter was deemed to be related to an error in the data extraction tool. Moreover, in 125 (28.3%) of the 441 episodes of care with falls, the reviewers considered that the falls, according to its definition, included an injury. All the falls with an injury in this group were classified in the review process to be minor, such as moderate pain, hematoma/swelling or abrasions.

In the EPRs where the predefined answer “Has fallen with injury” was used (group E), according to the data extraction tool, 15.4% (26/169) of the records, no falls could be found in the review, and in nine (34.6%) of these, the RNs correctly had documented in the fall term that there was no fall present during the episode of care. The latter was deemed to be related to errors in the data extraction tool. Also, in 17 (11.9%) of the 143 episodes of care with falls, the reviewers deemed that the falls did not include an injury.

**Fig. 1:**
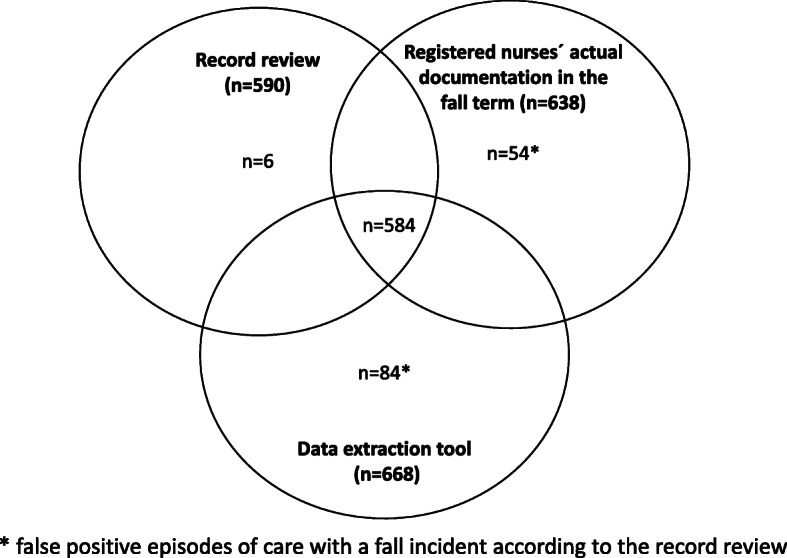
Episodes of care with at least one fall captured via the three data detection methods

Comparing the three data detection methods: the record review, the data extraction tool, and the RNs´ actual documentation of the presence of any fall in the episodes of care, for all sample groups are shown in Fig. 1. In summary, there were 584 out of 590 episodes of care with falls that were found with all three methods, and six falls were found only in the record review.

### Accuracy in documentation of falls

The positive predictive value in total for the data extraction tool regarding presence of any fall, in the respective episode of care, irrespectively of injury or not, in the groups D and E, in comparison with the record review outcome, was 87.4%. When the 27 episodes of care, correctly documented by the RNs, but by the data extraction tool incorrectly selected, had been withdrawn - the positive predictive value rose to 91.5%, see Table 3.

**Table 3:** Positive predictive values regarding presence of any fall in the respective episodes of care in the sample groups D and E

	PPV, %
**Selected via data extraction tool (*n*** **= 169)**	
D - Falls without injury according to the term	88.4
E - Falls with injury according to the term	84.6
Total	87.4
**Corrected selection based on the registered nurses´ actual documentation (*n*** **= 143)**	
D - Falls without injury according to the term	91.9
E - Falls with injury according to the term	90.5
Total	91.5

### Accuracy of registered nurses´ documentation of falls with non-injuries and injuries

The positive predictive value of the RNs´ documentation regarding presence of a fall in the respective episode of care, *without any injury* in the group D and *with at least one injury* in the group E in comparison with the record review outcome, was 71.7 and 88.1%, respectively.

### Potential errors

In total, 12.6% (*n* = 84) of the episodes of care in the groups D and E included a potential error (Table 4). The most common error was that the RNs documented that the patient had a fall in the fall term but no documented evidence for that could be found when reviewing the respective episode of care (*n* = 30, 25 from group D and five from group E). The second most common error was that the nurses correctly documented that no fall had occurred during the respective episode of care, but there was an error in the data extraction tool (*n* = 27, 18 from group D and nine from group E). When analyzing the latter, we found that this incorrect selection took place when the patient was transferred from one department to another within the hospital on the same day, and/or, for example, if the RN at the discharging ward activated the discharge note after the patient had been registered at the admitting ward. The data extraction tool then picked up information from the previous episode of care instead of the present.

**Table 4:** Potential causes of error regarding fall outcome in the groups D and E

Potential causes of error	Number (%) of episodes of care with a potential error
1. The registered nurses documented a fall in the fall term in the nursing discharge note but no documented evidence for that could be found during the respective episode of care when reviewing the record	30 (4.5)
2. The registered nurses correctly documented that no fall had occurred during the respective episode of care, i.e. it was an error in the data extraction tool	27 (4.0)
3. The registered nurses documented a fall that occurred at an episode of care just before the sampled one, i.e. the patient was still in the hospital, but it was another episode of care before the current	8 (1.2)
4. The registered nurses documented a fall that occurred before arrival to the hospital	6 (0.9)
5. The registered nurses had chosen two alternatives in the fall term, both fall without injury and no fall, none of these patients had any fall	6 (0.9)
6. The registered nurses had included fall at the emergency department that is not part of the current episode of care	3 (0.4)
7. The episode of care did not have a discharge note and the extraction tool took information from the direct previous episode of care	3 (0.4)
8. Fall in the patient’s own home during leave (still admitted)	1 (0.1)
Total	84 (12.6)

The total number of inpatient falls reported and confirmed in the clinical incident reporting system, for the same period and the same departments, were 439 compared to 714 falls detected through record review.

Concerning the presence of a fall during the sampled episode of care, the inter-rater reliability between the reviewers was classified as very good (*k =* 0.969).

## Discussion

In this study, the documentation of falls was reviewed in the EPR from time of admission until discharge in an extensive number of episodes of care at one major metropolitan area hospital. We found high accuracy in the RNs´ documentation at discharge on this important quality indicator – whether the patient had fallen or not during the episode of care. In more than 90% of the episodes of care amongst the “fallers” (groups D and E), the documentation at discharge was correct, compared to 87% for the outcome in the data extraction tool. The corresponding for the “non-fallers” was 100% for both the RNs and the data extraction tool (group C). In groups A and B, where there was no information on falls at discharge, falls were found in only three episodes of care, respectively. Furthermore, only 98 (group A) out of 31,571 episodes of care during the sampled year did not have any discharge notes from the RNs. The RNs´ documentation at discharge about whether the patient was injured or not, was, however, somewhat less valid. These were deemed by the reviewers to be related to minor fall injuries. More falls were identified in this study compared to the incident reporting system.

Data collection methods and sources to compare fall outcomes differ between validation studies and, to the best of our knowledge, a similar study cannot be found. Methods used to validate falls and/or fallers include text mining followed by record review versus the incident reporting systems [16], diagnostic codes found in routinely collected administrative hospitalization data versus record review [17], nurses´ estimates versus fall incident reports [18], participants’ self-report, participants’ case notes versus the hospital reporting systems [13], incident information management system versus the health information exchange using diagnostic codes [19], record review versus the hospital’s formal registry of adverse events [20], and a fall evaluation service versus incident reports [21]. In most studies, in concurrence with our study, the incident reporting systems were found not to be accurate to identify fall.

Despite knowledge of the shortcomings [10–12], in-hospital fall rate measurements are often based on incident reporting systems. Instead, we chose to review the records in a structured way using a review protocol and use this as the golden standard in this study. Different structured record review methodologies have been used extensively worldwide to explore different kinds of hospital-acquired events such as falls, infections, and pressure ulcers [22, 23] and have been proven to identify more events compared to, for example, incident reporting systems [10–12]. In Sweden, as in many other countries, all healthcare professionals are required by law to report incidents and yet our result showed a large discrepancy between the fall numbers identified in the EPR and the incident reporting system. This result is in accordance with other studies [13, 16, 19, 21] and confirms that incident reporting systems do not provide a valid measure of in-hospital falls. There are several reasons why professionals do not report incidents, such as falls. Barriers like extra work, skepticism, code of silence, fear of reprisals, lack of knowledge, uncertainty as to what constitutes an incident, or lack of effectiveness of present reporting systems, are only a few of the identified inhibitory reasons for this [24]. Nurses tend to report more incidents compared to physicians, but on the other hand, physicians report more serious, but not lethal, incidents [25]. Noble and Pronovost [26] argue that such participation bias hinders the ability to identify and reduce specific risks mostly viewed by physicians and may misdirect interventions. This might also give an incorrect picture of incidents occurring in different care processes. Haines et al. [27] investigated agreement between hospital staff on what constitutes a fall using video scenarios, as well as what incidents should be reported. They found that staff disagreed on several scenarios, both regarding what should be categorized as a fall and what should be reported as an incident. Fortunately, we did not find any falls when reviewing group C, and this was correct in 100% of cases for both the RN documentation and the data extraction tool.

It is advocated to combine various data sources identifying incidents, for example, falls [10, 11, 16, 28]. However, when different data collection methods are used either in combination or alone, the user must be aware of the respective method’s advantages and disadvantages when the data is interpreted. With the promising results from the present study, a different approach may be suggested, i.e., to use automated clinical nursing fall data from the EPR as the primary data source for collecting information on falls. Using already existing data saves time and enables instant and easy access to total population data and is also less susceptible to selection bias. Using automated data is also less resource-demanding for staff compared to, for example, cross-sectional measures, which reflect occasional snapshot data, and are often used in Sweden today to measure quality and safety. There are, however, challenges in using automated data from the EPR, such as the dependency of secure IT-technique and of standardized methods for accurate documentation [29]. As revealed in this present study, programming may not be specific enough due to overlapping time settings of the beginning and end of an episode of care. For instance, when a patient is transferred to a different ward and the new episode of care begins the same date, the information on falls extracted by the data extraction tool from the discharge note will “belong to” both episodes of care as they overlap in time. Moreover, the method requires documentation to be standardized so the specific fall term is always used and to use its standardized answers on each patient, as well as to assess injuries in accordance with its definition.

What were deemed minor injuries by the reviewers, caused by the fall, tended to be categorized by the RNs as “falls without injury” in more than one-quarter of the episodes of care in group D. Contributing factors to this may include personal attitude and knowledge to what is considered an injury or not, as well as the lack of use of a structured severity scale [30]. In the present study, we applied a modified version of the severity scale [15] used in the structured record review method named the Global Trigger Tool [31]. A severity scale may help the RNs to classify the falls that occur. Adverse event studies using a severity scale have found that the greatest variability between reviewers’ severity categorization was found among the cases with minor harm [32, 33]. The distinction between a fall without an injury and a fall with a minor injury is not sharp and is therefore subject to the respective RN’s assessment, which may affect documentation, and thereby, the fall severity outcome. Falls that lead to minor injury or no injury in one case might be related to nursing or organizational defects that could result in a more severe injury in another time or patient. Patient safety interventions are therefore needed to reduce all kinds of falls as well as to educate nursing staff regarding minor injuries.

As mentioned, the data extraction tool is dependent on the accuracy of the RNs´ documentation. A well-kept patient record is a basis for evaluation and follow-up in clinical health care. It can improve the care of, not only the individual patient, but also of its´ peers. Fifty-four of the 84 errors were related to deficiencies in the RNs´ documentation, such as when the RNs had included falls that happened elsewhere, either at another episode of care at the hospital or in the patient’s home before arrival to the hospital. The quality of the introduction of new RNs is therefore of great importance as well as discussions regarding falls and documentation.

Although the results of this study show good validity of the RNs’ documentation, minor adjustments could make it even better. The EPR ought to be as intuitive as possible (for example, it could automatically direct the user to select one of the predefined answers). Today, the EPR accepts any answer, either the predefined or free text answers. Thereby it is possible to “fool the system” by merely texting a dot in the free text box. Also, it should not be possible to select more than one answer, a flaw sometimes caused by a slip of the finger.

The programming in the data extraction tool has improved since 2016 to include “any note made*”* on this specific term “fall during episode of care”. This means that it captures information on falls independent of the professional category if they use the fall term in their notes. This is independent of when the documentation on the fall term is made during the episode of care and includes not only RNs´ documentation or the discharge note, but also in progress notes. If the patient has fallen more than once, the worst outcome of the fall is automatically noted.

### Strengths and limitations

Strengths of this study include that we chose to have a stringent definition of what constituted a fall and included all professionals´ notes in the review. This study has been conducted at a single site in a well-resourced healthcare system and we have had good control over the collection of patient data. To ensure the reliability and validity of our data, the study has been closely monitored to check the correctness of the data and discussions have been held within the research group on a regular basis. Furthermore, double reviews have been carried out with very good kappa value. Therefore, the data presented can be considered as very reliable. Another strength is that the hospital where the patients were included is a large acute public hospital representative of the routine care in the Swedish healthcare system; however, generalizations are made with caution.

There are also limitations connected to the study. To review all episodes of care (*N* = 31,571) during the inclusion period had been too time consuming. Therefore, randomization of the groups where falls were less expected to occur (B and C) was used. With this stratified selection procedure, about 4% of all episodes of care were reviewed.

When using record review as a data collection method, only documented incidents, in this case, falls, can be identified. This may lead to the number of falls and the positive predictive being underestimated for the RNs documentation. This is because, even if no documented evidence of a fall could be found when reviewing the records, the RN who documented in the discharge note may have had knowledge about the patient that had only been verbally exchanged between professionals.

Unfortunately, we were not able to follow all individual patients in the clinical incident reporting system, to match our sample groups, since the personal identity number is not an obligatory variable in the incident reporting system which could have been used to match the fall to the right episode of care. However, we were able to compare the outcome on an aggregate level for the included departments during the same inclusion period.

## Conclusions

Provided that adequate implementation strategies are given, the use of a specific term in the RNs´ discharge note regarding inpatient falls seems to be a valid and reliable measurement of falls in inpatient care. This may be used continuously to evaluate and follow-up nursing care on a total population.

## Data Availability

The datasets generated and/or analysed during the current study are not publicly available due to ongoing analysis addressing other research questions, but are available from the corresponding author on reasonable request.

## Abbreviations

EPR: Electronic Patient Record
IT: Information Technology
RN: Registered Nurse
